# Noninvasive oxygenation strategies in adult patients with acute respiratory failure: a protocol for a systematic review and network meta-analysis

**DOI:** 10.1186/s13643-020-01363-0

**Published:** 2020-04-26

**Authors:** Bruno L. Ferreyro, Federico Angriman, Laveena Munshi, Lorenzo Del Sorbo, Niall D. Ferguson, Bram Rochwerg, Michelle J. Ryu, Refik Saskin, Hannah Wunsch, Bruno R. da Costa, Damon C. Scales

**Affiliations:** 1 Interdepartmental Division of Critical Care Medicine, University of Toronto, Toronto, ON Canada; 2 Institute of Health Policy, Management and Evaluation, Dalla Lana School of Public Health, University of Toronto, Toronto, Canada; 3 Department of Medicine, Sinai Health System and University Health Network, Toronto, Canada; 4 Department of Critical Care Medicine, Sunnybrook Health Sciences Centre, Toronto, Canada; 5 Division of Respirology, Department of Medicine, University Health Network and University of Toronto, Toronto, Canada; 6 Department of Medicine, Division of Critical Care, McMaster University, Hamilton, Canada; 7 Department of Health Research Methods, Evidence, and Impact, McMaster University, Hamilton, ON Canada; 8 Sidney Liswood Health Sciences Library, Sinai Health System, Toronto, Canada; 9 Institute for Clinical Evaluative Sciences, Toronto, Ontario Canada; 10 Applied Health Research Center (AHRC), Li Ka Shing Knowledge Institute, St. Michael’s Hospital, Toronto, Canada; 11 Institute of Primary Health Care (BIHAM), University of Bern, Bern, Switzerland

**Keywords:** Hypoxia, Noninvasive ventilation, Oxygen, Oxygen inhalation therapy, Respiratory insufficiency

## Abstract

**Background:**

Acute hypoxemic respiratory failure is one of the leading causes of intensive care unit admission and is associated with high mortality. Noninvasive oxygenation strategies such as high-flow nasal cannula, standard oxygen therapy, and noninvasive ventilation (delivered by either face mask or helmet interface) are widely available interventions applied in these patients. It remains unclear which of these interventions are more effective in decreasing rates of invasive mechanical ventilation and mortality. The primary objective of this network meta-analysis is to summarize the evidence and compare the effect of noninvasive oxygenation strategies on mortality and need for invasive mechanical ventilation in patients with acute hypoxemic respiratory failure.

**Methods:**

We will search key databases for randomized controlled trials assessing the effect of noninvasive oxygenation strategies in adult patients with acute hypoxemic respiratory failure. We will exclude studies in which the primary focus is either acute exacerbations of chronic obstructive pulmonary disease or cardiogenic pulmonary edema. The primary outcome will be all-cause mortality (longest available up to 90 days). The secondary outcomes will be receipt of invasive mechanical ventilation (longest available up to 30 days). We will assess the risk of bias for each of the outcomes using the Cochrane Risk of Bias Tool. Bayesian network meta-analyses will be conducted to obtain pooled estimates of head-to-head comparisons. We will report pairwise and network meta-analysis treatment effect estimates as risk ratios and 95% credible intervals. Subgroup analyses will be conducted examining key populations including immunocompromised hosts. Sensitivity analyses will be conducted by excluding those studies with high risk of bias and different etiologies of acute respiratory failure. We will assess certainty in effect estimates using GRADE methodology.

**Discussion:**

This study will help to guide clinical decision-making when caring for adult patients with acute hypoxemic respiratory failure and improve our understanding of the limitations of the available literature assessing noninvasive oxygenation strategies in acute hypoxemic respiratory failure.

**Systematic review registration:**

PROSPERO CRD42019121755

## Background

Acute hypoxemic respiratory failure (AHRF) is among the leading causes of intensive care unit (ICU) admission in adult patients, [1], with around 40% of these patients receiving endotracheal intubation [2, 3]. Invasive mechanical ventilation (IMV) is associated with adverse events such as ventilator-associated pneumonia, ventilator-induced lung injury, and the potential for oversedation [4–6]. Avoiding unnecessary endotracheal intubation remains a major goal in the management of patients with AHRF [7–9]. Multiple noninvasive oxygenation strategies have been identified that might help avoid endotracheal intubation and decrease mortality, but it remains unclear which of these is most effective [9].

Previous studies have aimed to identify the optimal initial oxygenation strategy to avoid the receipt of invasive mechanical ventilation in adults with AHRF but have often produced conflicting results [10–14]. Classically, standard oxygen therapy (SOT) has been the conventional approach evaluated. Standard oxygen therapy provides low-flow oxygen (< 15 L/min) using different interfaces such as nasal prongs, Venturi system, and non-rebreather masks. In the early 2000s, noninvasive ventilation provided by a face mask (FM-NIV) was shown to be superior to SOT in two small single-center randomized controlled trials (RCT) [10, 11]. In 2015, a large RCT failed to show superiority of FM-NIV on the rates of endotracheal intubation and all-cause mortality compared to SOT in immunocompromised patients [12]. More recently, the use of high-flow nasal cannula (HFNC) gained interest and use among clinicians as an alternative method to provide noninvasive oxygenation [15–17]. A large 3-arm RCT recently compared the efficacy of HFNC, FM-NIV, and SOT in adults with AHRF [13]. Although this study did not show a benefit in the primary outcome (invasive mechanical ventilation at 28 days), HFNC was associated with decreased all-cause mortality at 90 days. Furthermore, a post hoc subgroup analysis showed that in patients with severe hypoxemia (PaO_2_:FiO_2_ < 200 mmHg) HFNC decreased the need of invasive ventilation when compared with the other two modalities. Notably, the highest mortality was seen among the FM-NIV treatment arm. This study started to raise concerns for potential harmful effects associated with FM-NIV, which were further supported by two large observational studies [18, 19]. Potential mechanisms of harm attributable to FM-NIV as compared to HFNC include the poor tolerance of the face-mask interface, increased risk of patient-ventilator asynchrony, and the inability to limit tidal volumes leading to high transpulmonary pressures and ventilator- and patient-inflicted lung injury [20–22]. As a result, recent guidelines have expressed uncertainty regarding the use of FM-NIV for the treatment of de novo AHRF [7]. Interestingly, in a subsequent study comparing HFNC vs SOT among immunocompromised patients, there were no differences noted in rates of invasive mechanical ventilation or mortality [14].

The use of a helmet interface to provide NIV (H-NIV) poses an attractive alternative to provide both oxygenation and ventilation in AHRF, potentially with better patient tolerability, patient-ventilator interaction, and the ability to provide ventilation for longer periods of time [23]. The potential benefits of H-NIV compared to SOT and FM-NIV to decrease the risk of ETI has been supported by recent RCTs [24–27]. In an RCT study conducted in 2010, H-NIV was found to decrease mortality and need for invasive mechanical ventilation compared to SOT in patients with hematologic malignancy [26]. Helmet-NIV was also found to decrease rates of invasive mechanical ventilation and mortality in a more recent single-center RCT compared to FM-NIV [25]. There are a series of physiologic mechanisms that may explain these results—predominantly related to the helmet interface. The helmet interface may act to decrease air leaks when compared to the face mask interface. This may permit more effective delivery of higher levels of positive end-expiratory pressure potentially increasing alveolar recruitment and decreasing respiratory drive [2, 8, 22, 27]. Furthermore, patients with AHRF seem to have better tolerance for a helmet interface minimizing the time that the therapy requires interruption—something not uncommon for FM-NIV [25]. Interestingly, there are currently no available head to head comparisons of H-NIV and HFNC for relevant clinical outcomes such as mortality and need for invasive mechanical ventilation.

Determining the optimal strategy to decrease rates of endotracheal intubation in patients with AHRF is essential to minimize morbidity and mortality associated with the need for invasive mechanical ventilation. Although HFNC has become widely used, evidence is lacking regarding its effect as compared to standard oxygen, and there are no head to head comparisons between HFNC and H-NIV. The limitations of the current evidence are clearly reflected in clinical practice, where there exists high variability among clinicians in the use of these four noninvasive oxygenation strategies for AHRF [8]. Furthermore, although previous systematic reviews and meta-analyses have explored the effectiveness of HFNC in patients with AHRF, these studies have often pooled control group data as “any other strategy,” failing to account for the important physiologic and clinical differences of these alternative interventions [28–30].

Network meta-analysis (NMA) is a strategy to summarize the evidence of clinical studies which include multiple treatments [31]. By incorporating estimates from direct comparisons and indirect comparisons, NMAs allow estimation of treatment effects where head to head comparisons are scarce and can decrease the imprecision in pooled treatment effects even when head to head comparisons exist [32]. To our knowledge, no previous NMAs have been conducted to compare the relative effectiveness of all available noinvasive oxygenation strategies among a general population of adult patients with AHRF.

The primary objective of this NMA is to assess the association between different noninvasive oxygenation strategies and all-cause mortality in adult patients with AHRF. The secondary objective is to assess the association between different noninvasive oxygenation strategies and the receipt of invasive mechanical ventilation in adult patients with AHRF.

## Methods

This protocol has been written in accordance with the Preferred Reporting Items for Systematic Review and Meta-Analysis Protocols 2015 statement (PRISMA-P) and its extension for NMA [33, 34]. This protocol is also registered in the International Prospective Register of Systematic Reviews (PROSPERO, CRD42019121755).

### Criteria for included studies

#### Participants and settings

We will include any studies that enrolled adult patients (18 years of age or older) with AHRF, defined by the new onset of clinical signs (tachypnea, increased work of breathing), radiologic signs (unilateral or bilateral chest x-ray opacities), and gas exchange alterations (different degrees of hypoxemia). We will consider studies including patients treated in the intensive care unit, intermediate care unit, medical wards, and emergency department. Studies primarily focused in the treatment of acute exacerbations of chronic obstructive pulmonary disease (AE-COPD) or congestive heart failure (CHF) will be excluded. Specifically, we will exclude such studies if 50% or more of the included population are patients with either AE-COPD or CHF, acknowledging that a lower threshold will exclude too many studies in the field. Our pre-planned sensitivity analysis will specifically address this issue. The rationale for exclusion is based in the already shown superiority of noninvasive ventilation for these conditions. Furthermore, we will also exclude studies including patients after extubation for major thoracic surgery.

#### Interventions and comparators

All interventions considered in this review are noninvasive oxygenation strategies. HFNC oxygenation refers to oxygen delivered through a nasal cannula, at a FIO_2_ up to 1.0 and a flow rate that can be as high as 60 l/min. NIV provides positive pressure ventilation that can be delivered with different modalities: continuous positive airway pressure, bi-level positive airway pressure, or pressure support ventilation. The interfaces used can be either a face mask (FM-NIV) or a helmet (H-NIV). Given the potential differences in reported outcomes, these interfaces will be considered as two different interventions in the network. SOT (low-flow systems) comprise traditional nasal cannula, Venturi system masks, or non-rebreather masks. All of these modalities can provide oxygen at low flow (< 15 l/min) with varying levels of FIO_2_.

#### Outcomes measures

The primary outcome of this study will be all-cause mortality, defined as the longest available in the first 90 days after randomization. The secondary outcome will be receipt of invasive mechanical ventilation (longest available at 30 days). Given that the criteria for endotracheal intubation might vary between studies, we will collect information on whether decisions to intubate were based on pre-specified criteria or not.

#### Study design and publication types

We will include only randomized controlled trials focused on the treatment of acute hypoxemic respiratory failure if a combination of any of these interventions was assessed: HFNC, FM-NIV, H-NIV, and SOT.

### Information sources and search strategy

The following seven electronic bibliographic databases will be searched using a comprehensive search strategy developed by an information specialist (MR): (1) *Ovid MEDLINE*, (2) *Ovid EMBASE*, (3) *PubMed* (*non-MEDLINE records only*), (4) *Ovid EBM Reviews*-*Cochrane Central Register of Controlled Trials*, (5) *EBSCO CINAHL Complete*, (6) *Web of Science*, and (7) *LILACS*. We will also search *ClinicalTrials*.*gov*, *WHO International Clinical Trials Registry Platform*, and *International Standard Randomized Controlled Trial Number Registry* for all registered clinical trials and randomized controlled trials. The search strategy will be structured according to the *Peer Reviewed Electronic Search Strategies* (*PRESS*) *2015 Guidelines* [35] (refer to Supplementary File for full search strategy). We will use a validated search filter for randomized controlled trials from the *Cochrane Handbook for Systematic Reviews of Interventions Version 5*.*1*.*0*, *Section 6*.*4*.*11* [36] to screen *Ovid MEDLINE*, *Ovid EMBASE*, and *PubMed*. We will adapt a pre-tested search filter for randomized controlled trials from the *Scottish Intercollegiate Guidelines Network* [37] for *EBSCO CINAHL Complete* and *Web of Science*. No limits will be applied to language, publication year, gender or race. We will manage all references and duplicates using EndNote X8 citation management software.

### Study selection

Two reviewers (BLF and FA) will screen independently the titles and abstracts retrieved from the search strategy and the additional sources in order to identify those meeting the mentioned eligibility criteria. Subsequently, we will obtain full texts of the articles meeting these pre-specified criteria and review again in a second stage. Any disagreement between the reviewers will be discussed and referred to final decision by a third investigator (LM, HW, DS).

### Data extraction

The two reviewers (BLF and FA) will perform data extraction independently in a pre-piloted data extraction form created in Excel (Microsoft®). Any differences will be resolved by consensus or discussion with a third author (LM). Abstracted data will include study characteristics (trial design, size, and funding source), patients’ characteristics (age, sex, etiology of AHRF, immunocompromised status, and the presence of severe AHRF (PaO_2_:FIO_2_ ratio < 200)), details of the interventions (location of application, duration of the exposure to each of the oxygenation strategy, type of noninvasive ventilation modality), outcome data for each endpoint of interest.

### Risk of bias assessment

The risk of bias will be assessed independently using the tools specified in the Cochrane Handbook for Systematic Reviews of Interventions for RCTs [27–30]. The following aspects will be assessed: (1) random sequence generation (selection bias), (2) allocation concealment (selection bias), (3) blinding of participants and personnel (performance bias), (4) blinding of outcome assessment (detection bias), (5) incomplete outcome data, (6) selective reporting (reporting bias), and (7) other sources of bias.

### Patient and public involvement

This research will be done without patient involvement. Patients will not be invited to comment on the protocol design nor consulted to develop patient-relevant outcomes.

### Data synthesis and analysis

We will summarize the included studies based on study and patient characteristics, outcome measures, and risk of bias. We will perform a series of pairwise Bayesian meta-analyses with a random-effects model, followed by a network meta-analysis using a Bayesian framework to derive head-to-head treatment effect estimates comparing all interventions. Analyses will be based on Markov chain Monte Carlo methods using prior distributions for event rates derived from previous literature, minimally informative treatment effect estimates, and informative prior distributions for heterogeneity estimates derived from external evidence [38]. We will report pairwise and NMA treatment effect estimates as risk ratios (RR), estimating summary treatment effect estimates from the median and corresponding 95% credible intervals (CrIs) from the 2.5th and 97.5th percentile of the posterior distribution. We will also rank interventions according to their apparent effectiveness and will calculate the probability that interventions have risk reductions or increases greater compared to standard oxygen therapy. We will quantify heterogeneity in treatment effects between studies using the posterior distribution *τ*^2^. Inconsistency (incoherence) between direct and indirect comparisons will be estimated using the node-splitting approach contrasting estimates from both direct and indirect evidence [39, 40]. We will visually assess model convergence using the Brooks-Gelman-Rubin diagnostic, trace plots, and auto-correlation plots. We will assess the goodness-of-fit of our final models by comparing the mean residual deviance with the number of contributing data points, calculating the percentage of standardized node-based residuals within 1.96 of the standard normal distribution, and visually inspecting the distribution of residuals on Q-Q plots. We will perform all analyses in R v3.6 and OpenBUGS.

### Assessment of publication bias

We will assess for the presence of publication bias by examining the comparison-adjusted funnel plot [41]. We will examine the shape of the funnel plot and assume that there is a risk of publication bias if its shape is asymmetrical. We will formally test for asymmetry in the funnel plot by performing the Harbord’s test [42].

### Subgroup analysis

Where information is available, we will assess for credible subgroup effects using a random-effects NMA meta-regression Bayesian model to determine whether the estimated treatment effects are affected by the following factors: (1) severe AHRF (PaO_2_:FIO_2_ ratio < 200) vs less severe AHRF (PF ratio > 200), hypothesizing that HFNC is better than SOT for patients with severe AHRF, and (2) immunocompromised patients (hematologic malignancies, solid tumors with active chemotherapy, treatment with immunosuppressant drugs, and solid organ transplant recipients) vs non-immunocompromised patients, hypothesizing that NIV may be more effective in immunocompromised patients.

### Sensitivity analysis

We will perform sensitivity analysis for the treatment effect estimates by excluding studies with high risk of bias in each of the assessed items. Furthermore, we will conduct a sensitivity analysis by excluding those studies that included any patients with AE-COPD or CHF, and with non-informative prior distributions for the heterogeneity parameters.

### Grading of recommendations

We will rate the quality of each direct, indirect, and NMA estimates based on the four-step approach suggested by the Grading of Recommendations Assessment, Development and Evaluation (GRADE) Working Group [43]. The network effect estimates for each of the potential comparisons and every outcome, along with the GRADE rating for certainty in the evidence, will be depicted in a summary of findings table.

## Discussion

In summary, this Bayesian network meta-analysis will provide a comprehensive summary of the direct and indirect evidence on the efficacy of different noninvasive oxygenation strategies to prevent mortality and the receipt of invasive mechanical ventilation in adult patients with acute hypoxemic respiratory failure. The current protocol was written in accordance with the Preferred Reporting Items for Systematic Review and Meta-Analysis Protocols 2015 statement (PRISMA-P) and its extension for NMA. This protocol is also registered in the International Prospective Register of Systematic Reviews (PROSPERO). The overall quality of the evidence will be assessed using the Grading of Recommendations Assessment Development and Evaluation (GRADE) approach.

We expect this study will help with clinical decision-making when caring for adult patients with AHRF. Furthermore, the systematic approach will improve our understanding of the limitations of the available literature to inform the treatment of patients with AHRF. Finally, we believe that our results will help identify predictive enrichment strategies that may improve the design of future randomized controlled trials.

## Supplementary information

**Additional file 1.** NMA-CRFRI.

## Data Availability

Data sharing is not applicable to this article as no datasets were generated or analyzed during the current protocol.

## Abbreviations

AHRF: Acute hypoxemic respiratory failure
ICU: Intensive care unit
IMV: Invasive mechanical ventilation
SOT: Standard oxygen therapy
FM-NIV: Face mask noninvasive ventilation
RCT: Randomized controlled trial
HFNC: High-flow nasal cannula
H-NIV: Helmet noninvasive ventilation
NMA: Network meta-analysis
AE-COPD: Acute exacerbation of chronic obstructive pulmonary disease
CHF: Congestive heart failure

## References

[1] Scala R, Heunks L (2018). Highlights in acute respiratory failure. Eur Respir Rev.

[2] Nava S, Hill N (2009). Noninvasive ventilation in acute respiratory failure. Lancet.

[3] de Jong A, Calvet L, Lemiale V, Demoule A, Mokart D, Darmon M, et al. The challenge of avoiding intubation in immunocompromised patients with acute respiratory failure. Expert Review of Respiratory Medicine 2018;0(0):1-14. 10.1080/17476348.2018.1511430 30101630

[4] Strøm T, Toft P (2014). Sedation and analgesia in mechanical ventilation. Semin Respir Crit Care Med.

[5] Slutsky AS, Ranieri VM (2013). Ventilator-induced lung injury. N Engl J Med.

[6] Kalanuria AA, Ziai W, Mirski M (2014). Ventilator-associated pneumonia in the ICU. Crit Care.

[7] Rochwerg B, Brochard L, Elliott MW, Hess D, Hill NS, Nava S, et al. Official ERS/ATS clinical practice guidelines: noninvasive ventilation for acute respiratory failure. European Respiratory Journal. 2017;50(2). 10.1183/13993003.02426-2016 28860265

[8] Garcia-de-Acilu M, Patel BK, Roca O (2019). Noninvasive approach for de novo acute hypoxemic respiratory failure: noninvasive ventilation, high-flow nasal cannula, both or none?. Curr Opin Crit Care.

[9] Dugan KC, Hall JB, Patel BK (2018). High-flow nasal oxygen-the pendulum continues to swing in the assessment of critical care technology. JAMA.

[10] Hilbert G, Gruson D, Vargas F, Valentino R, Gbikpi-Benissan G, Dupon M (2001). Noninvasive ventilation in immunosuppressed patients with pulmonary infiltrates, fever, and acute respiratory failure. N Engl J Med.

[11] Antonelli M, Conti G, Bufi M, Costa MG, Lappa A, Rocco M (2000). Noninvasive ventilation for treatment of acute respiratory failure in patients undergoing solid organ transplantation: a randomized trial. JAMA.

[12] Lemiale V, Mokart D, Resche-Rigon M, Pène F, Mayaux J, Faucher E (2015). Effect of noninvasive ventilation vs oxygen therapy on mortality among immunocompromised patients with acute respiratory failure. JAMA.

[13] Frat J-P, Thille AW, Mercat A, Girault C, Ragot S, Perbet S (2015). High-flow oxygen through nasal cannula in acute hypoxemic respiratory failure. N Engl J Med.

[14] Azoulay E, Lemiale V, Mokart D, Nseir S, Argaud L, Pene F (2018). Effect of high-flow nasal oxygen vs standard pxygen on 28-day mortality in immunocompromised patients with acute respiratory failure: the HIGH randomized clinical trial. JAMA.

[15] Goligher EC, Slutsky AS (2017). Not just oxygen? Mechanisms of benefit from high-flow nasal cannula in hypoxemic respiratory failure. Am J Respir Crit Care Med.

[16] Coudroy R, Jamet A, Petua P, Robert R, Frat J-P, Thille AW (2016). High-flow nasal cannula oxygen therapy versus noninvasive ventilation in immunocompromised patients with acute respiratory failure: an observational cohort study. Ann Intensive Care.

[17] Nishimura M (2015). High-flow nasal cannula oxygen therapy in adults. J Intensive Care.

[18] Bellani G, Laffey JG, Pham T, Madotto F, Fan E, Brochard L (2017). Noninvasive ventilation of patients with acute respiratory distress syndrome. Insights from the LUNG SAFE study. Am J Respir Crit Care Med.

[19] Carteaux G, Millán-Guilarte T, De Prost N, Razazi K, Abid S, Thille AW (2016). Failure of noninvasive ventilation for de novo acute hypoxemic respiratory failure: role of tidal volume. Crit Care Med.

[20] Brochard L, Slutsky A, Pesenti A (2017). Mechanical ventilation to minimize progression of lung injury in acute respiratory failure. Am J Respir Crit Care Med.

[21] Carteaux G, Lyazidi A, Cordoba-Izquierdo A, Vignaux L, Jolliet P, Thille AW (2012). Patient-ventilator asynchrony during noninvasive ventilation: a bench and clinical study. Chest..

[22] Grieco DL, Menga LS, Eleuteri D, Antonelli M (2019). Patient self-inflicted lung injury: implications for acute hypoxemic respiratory failure and ARDS patients on noninvasive support. Minerva Anestesiol.

[23] Esquinas Rodriguez AM, Papadakos PJ, Carron M, Cosentini R, Chiumello D (2013). Clinical review: helmet and noninvasive mechanical ventilation in critically ill patients. Critical care (London, England).

[24] Brambilla AM, Aliberti S, Prina E, Nicoli F, Del Forno M, Nava S (2014). Helmet CPAP vs. oxygen therapy in severe hypoxemic respiratory failure due to pneumonia. Intensive Care Med.

[25] Patel BK, Wolfe KS, Pohlman AS, Hall JB, Kress JP (2016). Effect of noninvasive ventilation delivered by helmet vs face mask on the rate of endotracheal intubation in patients with acute respiratory distress syndrome. JAMA..

[26] Squadrone V, Massaia M, Bruno B, Marmont F, Falda M, Bagna C (2010). Early CPAP prevents evolution of acute lung injury in patients with hematologic malignancy. Intensive Care Med.

[27] 33rd Congress of the Scandinavian Society of Anaesthesiology and Intensive Care Medicine. Acta anaesthesiologica scandinavica. 2015;59. 10.1111/aas.12556 26059918

[28] Algamdi M, Ball I (2016). High flow nasal cannula oxygen therapy for acute hypoxemic respiratory failure: a systematic review. Chest.

[29] Lin S-M, Liu K-X, Lin Z-H, Lin P-H (2017). Does high-flow nasal cannula oxygen improve outcome in acute hypoxemic respiratory failure? A systematic review and meta-analysis. Respir Med.

[30] Sklar MC, Mohammed A, Orchanian-Cheff A, Del Sorbo L, Mehta S, Munshi L (2018). The impact of high-flow nasal oxygen in the immunocompromised critically ill: a systematic review and meta-analysis. Respir Care.

[31] Brignardello-Petersen R, Rochwerg B, Guyatt GH (2014). What is a network meta-analysis and how can we use it to inform clinical practice?. Pol Arch Med Wewn.

[32] Greco T, Biondi-Zoccai G, Saleh O, Pasin L, Cabrini L, Zangrillo A (2015). The attractiveness of network meta-analysis: a comprehensive systematic and narrative review. Heart Lung Vessel.

[33] Moher D, Shamseer L, Clarke M, Ghersi D, Liberati A, Petticrew M (2015). Preferred reporting items for systematic review and meta-analysis protocols (PRISMA-P) 2015 statement. Syst Rev.

[34] Hutton B, Salanti G, Caldwell DM, Chaimani A, Schmid CH, Cameron C (2015). The PRISMA extension statement for reporting of systematic reviews incorporating network meta-analyses of health care interventions: checklist and explanations. Ann Intern Med.

[35] McGowan J, Sampson M, Salzwedel DM, et al. PRESS Peer Review of Electronic Search Strategies: 2015 guideline statement. *Journal of Clinical Epidemiology*. 201;75:40-46. doi.org/10.1016/j.jclinepi.2016.01.021. 10.1016/j.jclinepi.2016.01.021 27005575

[36] Higgins JPT, Green S (editors). Cochrane handbook for systematic reviews of interventions Version 5.1.0 [updated March 2011]. The Cochrane Collaboration, 2011. Available from www.handbook.cochrane.org.

[37] Scottish Intercollegiate Guidelines Network (SIGN). Randomised controlled trials search filter. *Healthcare Improvement Scotland*. [undated]. Available from: https://www.sign.ac.uk/search-filters.html.

[38] Turner RM, Jackson D, Wei Y, Thompson SG, Higgins JP (2015). Predictive distributions for between-study heterogeneity and simple methods for their application in Bayesian meta-analysis. Stat Med.

[39] van Valkenhoef G, Dias S, Ades AE, Welton NJ (2016). Automated generation of node-splitting models for assessment of inconsistency in network meta-analysis. Res Synth Methods.

[40] Higgins JP, Jackson D, Barrett JK, Lu G, Ades AE, White IR (2012). Consistency and inconsistency in network meta-analysis: concepts and models for multi-arm studies. Res Synth Methods.

[41] Chaimani A, Higgins JP, Mavridis D, Spyridonos P, Salanti G (2013). Graphical tools for network meta-analysis in STATA. PLoS One.

[42] Harbord RM, Egger M, Sterne JA (2006). A modified test for small-study effects in meta-analyses of controlled trials with binary endpoints. Stat Med.

[43] Puhan MA, Schunemann HJ, Murad MH, Li T, Brignardello-Petersen R, Singh JA (2014). A GRADE working group approach for rating the quality of treatment effect estimates from network meta-analysis. BMJ..

